# 

**Published:** 1867-09

**Authors:** 


### CONTENTS.

### ORIGINAL CONTRIBUTIONS.

ART. XXXVIII. Cerebro-Spinal Meningitis. By F. R. Payne, M.D., Marshal, Ill. 513

ART. XXXIX. Experimental Inquiries Concerning the Physiological Effects of Alcoholic Drinks on Man. By N. S. Davis, M.D., Professor Practical and Clinical Medicine, Chicago Medical College. 522

ART. XL. Homœpathic Life Insurance. By S. R. Millard, M.D., of Aurora, Ill. 526

ART. XLI. Poisoning by Strychnine.—Recovery. By Stacy Hemenway, Eugene City, Oregon. 528

### PROCEEDINGS OF SOCIETIES.

Adams County Medical Society. 530

Quincy Medical Society. 535

### CORRESPONDENCE.

Ratio of Deaths from Chloroform in England. 538

Local Anæsthesia. 540

### SELECTIONS.

Diabetic Phthisis and its Treatment. 541

Chronic Metritis—Prof. Scanzoni's Treatment. 545

A case of Excision of the Cervix Uteri. 546

Diabetes. By H. Bence Jones, A.M., M.D., F.R.S., late Physician to St. George's Hospital. 548

Training, or Forced Exercise in the Treatment of Diabetes. 550

Diabetes. 552

Treatment of Diabetes. By Abbots Smith, M.D., M.R.C.P., &c. 552

Treatment of Incontinence of Urine. By Abbots Smith, M.D., M.R.C.P., &c. 554

### BOOK NOTICES.

The Physiology and Pathology of the Mind. By Henry Maudsley, M.D. 554

Essentials of the Principles and Practice of Medicine. By Henry Hartshorne, M.D. 556

The Principles and Practice of Disinfection. By Roberts Bartholew, M.D. 556

Prize Essay on Vital Statistics. By Franklin B. Hough, M.D. 556

Report on Epidemic Cholera. Circular No. 5. Surgeon Generals Office, War Department, May 4th, 1867 557

The Quarterly Journal of Psycological Medicine and Medical Jurisprudence. Edited by Wm. A. Hammond, M.D. 557

### EDITORIAL.

Duffield, Parke & Co. 557

Chicago Medical Society. 558

New Medical Journals. 560

Medical Education in Chicago. 560

Deaths. 561

Cook County Hospital. 561

Medical Organization in Canada. 563

New Sydenham Society. 564

Obituary.—Death of Dr. Orren Smith. 565

New York Medical Society on Consanguineous Marriages. 566

Clinical Instruction at the Chicago Eye and Ear Infirmary. 567

Mortality Report. 568

To Physicians. 569

